# ACMG/AMP‐Based Variant Classification of a Novel *HBA2* Variant ( *HBA2* : C.297del, Hb Taiping) in Compound Heterozygosity With Hb Adana ( *HBA2* :C.179G>A) Causing Non‐Deletional Hb H Disease

**DOI:** 10.1111/ijlh.70037

**Published:** 2026-01-06

**Authors:** Norafiza Mohd Yasin, Suguna Somasundram, Syahzuwan Hassan, Nur Aisyah Aziz, Faidatul Syazlin Abdul Hamid, Ezzanie Suffya Zulkefli, Nor Nazuha Mohamad, Chin Ming Lee, Mohd Nazif Darawi, Thessalia Papasavva, Coralea Stephanou, Petros Kountouris, Sandra J. G. Arkesteijn, Tamara Koopman, Cornelis L. Harteveld

**Affiliations:** ^1^ Haematology Unit, Cancer Research Centre (CaRC), Institute for Medical Research (IMR) National Institute for Health (NIH) Setia Alam Setia Alam Selangor Malaysia; ^2^ Department of Clinical Genetics/GD Leiden University Medical Centre Leiden the Netherlands; ^3^ Haematology Unit, Pathology Department Hospital Taiping Taiping Perak Malaysia; ^4^ Paediatric Unit, Department of Medicine Hospital Taiping Taiping Perak Malaysia; ^5^ Department of Medical Diagnostics, Faculty of Health Sciences University Selangor Shah Alam Selangor Malaysia; ^6^ Molecular Genetics Thalassaemia Department Cyprus Institute of Neurology and Genetics Nicosia Cyprus

**Keywords:** alpha‐thalassaemia, novel variant, variant classification

## Abstract

**Background:**

Accurate classification of novel globin gene variants is critical for the diagnosis and management of thalassaemia. The adaptation of ACMG/AMP guidelines for globin genes represents an important step toward standardising variant interpretation and enhancing clinical utility in the field. This study reports the haematological and molecular characteristics of a novel α2‐globin variant identified in a Malay family.

**Methods:**

A Malay family from Taiping, Perak, Malaysia, with a history of α‐thalassaemia, was recruited for this study. The proband's phenotype was assessed through comprehensive haematological analysis and clinical evaluation. Known α‐thalassaemia deletions and non‐deletional mutations were screened using gap‐PCR and ARMS‐PCR. Sanger sequencing of the *HBA* genes was conducted to characterise the proband's genotype in detail.

**Results:**

A novel pathogenic *HBA2* variant was identified, expanding the known mutational spectrum of α‐thalassaemia. This variant introduces a premature stop codon, occurs in trans with a known pathogenic allele associated with a significant clinical phenotype, segregates with the disease in the family, and is absent from major population databases. Based on haematological data, molecular findings, in silico predictions, and protein modeling, the variant meets the ACMG/AMP criteria for pathogenicity adapted for α‐globin genes. We have designated this variant Hb Taiping, named after the location of its discovery. Its accurate classification is vital for carrier screening, genetic counselling, and prenatal diagnosis, thereby supporting improved clinical management.

**Conclusion:**

This study identifies and characterises a novel α‐globin gene variant, Hb Taiping, in a Malay family with α‐thalassaemia. The discovery contributes to the growing body of pathogenic mutations linked to α‐thalassaemia and underscores the importance of precise variant classification for effective diagnosis, risk assessment, and genetic counselling.

## 1 Introduction

Thalassaemia is the most common inherited recessive haemoglobin (Hb) disorder globally and poses a significant public health burden, particularly in the Mediterranean region, the Middle East, India, Southeast Asia, and Africa. However, due to increasing migration, haemoglobinopathies are now emerging as public health concerns in non‐endemic, high‐income countries, including those in northern Europe.

The most severe form of α‐thalassaemia is Hb Bart's hydrops fetalis syndrome, which typically results in intrauterine or early neonatal death. In contrast, Hb H disease is a non‐fatal form of α‐thalassaemia that presents with a variable clinical spectrum ranging from mild to severe haemolytic anaemia, often accompanied by hepatosplenomegaly [1, 2]. Historically, the prevalence of Hb H disease was highest in Asia, the Mediterranean, and the Middle East due to the high allele frequencies of common deletions such as ‐‐^SEA^ and ‐‐^MED^. However, intermarriage and population migration have significantly altered the global distribution patterns of α‐thalassaemia [1, 3].

Compared to deletional Hb H disease, non‐deletional forms are generally more severe [4, 5]. In Southeast Asia, common non‐deletional α‐thalassaemia variants include Hb Constant Spring, Hb Adana, Hb Quong Sze, and Hb Pakse [6]. To date, more than 600 alpha gene variants have been described in two databases, HbVar (https://globin.bx.psu.edu/cgi‐bin/hbvar/counter) [7] and Ithanet Genes (https://www.ithanet.eu/) [8]. Few have been reported as variants that cause a premature stop codon in either the *HBA1* and *HBA2* gene.

In this study, we investigated a Malay family with α‐thalassaemia and identified a previously unreported *HBA2* variant. Through a combination of clinical phenotype analysis, molecular characterisation, in silico prediction tools, family segregation studies, and population frequency data from gnomADv4.1, we gathered strong evidence to classify this novel variant as likely pathogenic, based on ACMG/AMP guidelines adapted for α‐globin genes [9, 10].

## 2 Methods

**FIGURE 1:**
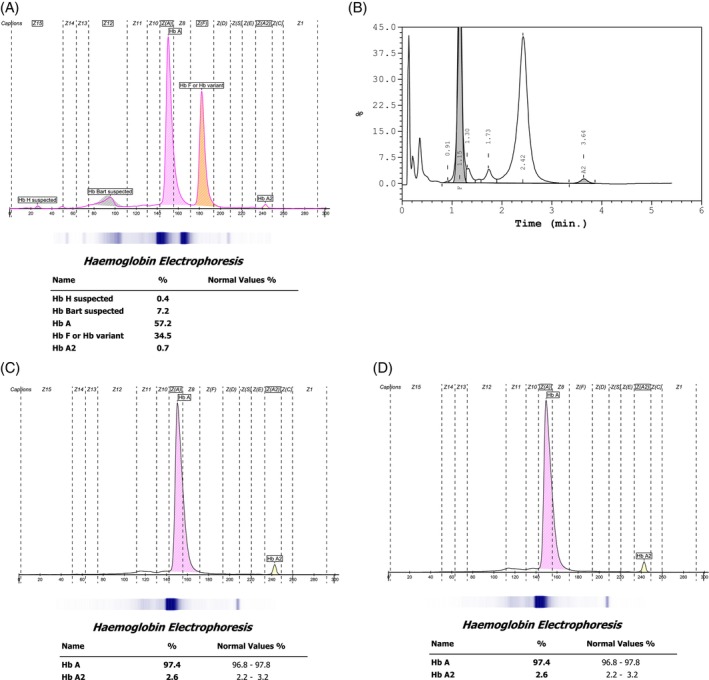
Haemoglobin Analysis of the Proband. Panels (A) and (B) show haemoglobin analysis results obtained using capillary electrophoresis (CE) and HPLC Variant II, respectively. The red arrow indicates the Hb H peak detected by CE (0.4%) and the pre‐calibration peak observed in the HPLC profile. Both methods revealed low HbA_2_ levels, with percentages of 0.7% (CE) and 1.3% (HPLC). Panels (C) and (D) display CE chromatograms from the proband's parents, both showing normal haemoglobin patterns.

**FIGURE 2:**
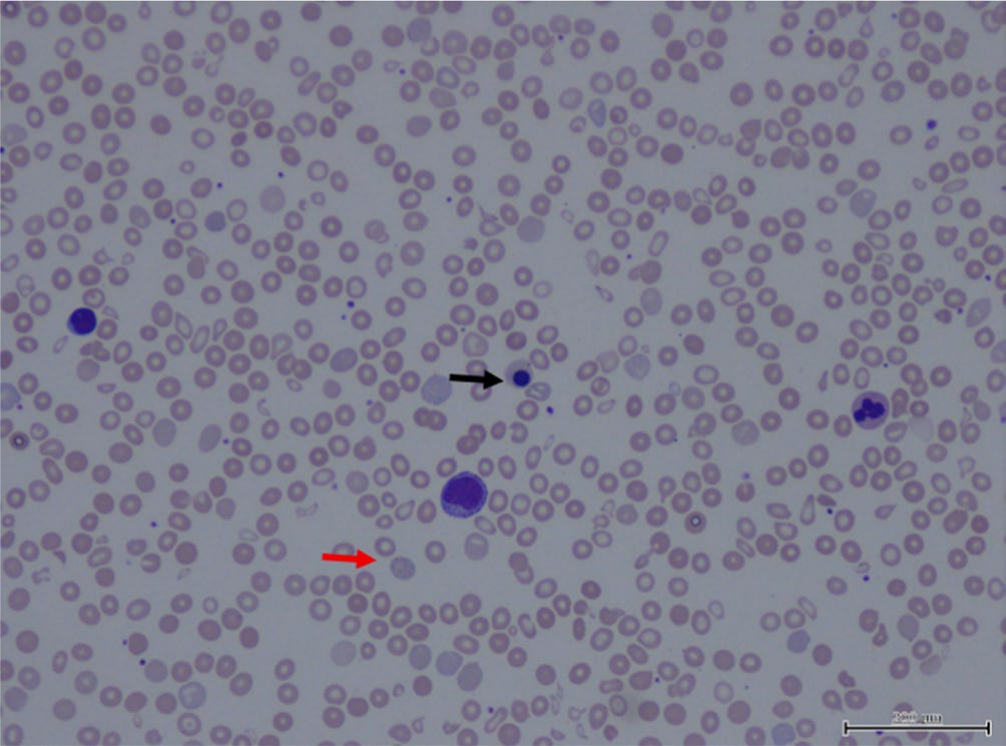
Blood film of the patient hypochromic microcytic anaemia with increased polychromasia and marked anisopoikilocytosis. Poikilocytes are include teardrop cells, target cells and fragments. There is also basophilic stippling (red arrow) and nucleated red cells (black arrow).

The proband, a 2‐year‐old Malay girl, was identified through family screening following positive α‐thalassaemia screening in both parents. She first presented at the age of 1 month with haemoglobin (Hb)of 10.2 g/dL, during a workup for prolonged neonatal jaundice. At 2 months, she was re‐admitted with Hb of 6.8 g/dL and marked microcytic hypochromia (MCV 71.4 fL, MCH 21.2 pg) respectively. Iron supplementation was initiated, and Hb analysis suggested Hb H disease (Figure 1). Peripheral blood film showed hypochromic microcytic anaemia with increased polychromasia and marked anisopoikilocytosis with few basophilic stippling and nucleated red cells (Figure 2). Due to logistical constraints, the family defaulted follow‐up. At the age of 2 years and 1 month, she received her first packed red cell transfusion (pre‐transfusion Hb 6.9 g/dL). Follow‐up remained inconsistent due to socioeconomic issues. At 2 years and 7 months, she re‐presented with pallor and reduced activity, her Hb was 5.5 g/dL, requiring transfusion. Between April 2024 and May 2025, the patient received five documented transfusions. Pre‐transfusion Hb levels ranged from 5.5 to 7.5 g/dL, indicating persistent moderate to severe anaemia. Though scheduled for 6‐weekly follow‐ups, actual intervals often extended to 10 weeks due to ongoing challenges.

### 2.1 Birth and Family History

The proband was born at 37 weeks' gestation via elective Caesarean section, due to a prior Caesarean and maternal requestwith a birth weight of 3.5 kg. She required brief postnatal care for transient tachypnoea and was discharged in good condition after 2 days. The antenatal period was uneventful.

She is the only child from her mother's second marriage. Both parents are 23 years old and non‐consanguineous. The mother has a 5‐year‐old daughter from a previous marriage who is reportedly healthy. There is no known family history of thalassaemia. Genetic testing revealed that the father carries a heterozygous *HBA2* mutation at codon 59 (Hb Adana, *HBA2*:c.179G>A). Initial screening of the mother using Gap‐PCR and ARMS‐PCR was negative for common alpha thalassaemia deletions and mutations. Subsequent sequencing identified a novel variant in the *HBA2* gene at codon 98, Hb Taiping (*HBA2*:c.297del), leading to a frameshift mutation. This variant has been submitted by our team to ITHANET database with IthaID: 4137.

Haematological profiles of the proband and her parents are summarised in Table 1.

**TABLE 1:** The haematological data, red cell indices, and α and β‐globin gene genotype of the proband, and her parents.

Parameters	Proband	Father	Mother
RBC (10^12^/L) M:4.5–5.5 × 10^12^/L F:3.8–4.8 × 10^12^/L	3.25 (4.6 ± 0.6)	6.51	5.67
Hb (g/dl) M: 13.0–17.0 g/dL F: 12.0–15.0 g/dL	6.9 (12.5 ± 15)	15.4	13.1
MCV (fL) (81–101 fL)	71.4 (81 ± 6)	77.6	70.9
MCH (pg) (27–32 pg)	21.2 (27 ± 3)	23.7	23.1
RDW‐CV (%)	25.2	13.6	15.8
CE (%)			
Hb A	—	—	—
Hb A2	0.7	2.6	2.6
Hb F	34.5	—	—
Hb variant	Hb H: 0.4%, Hb Bart's: 7.2	—	—
HPLC (%)		NA	NA
Hb A	—		
Hb A2	1.3		
Hb F	33.2		
Hb Variant	Pre‐run peak		
H‐Inclusion	Positive	NA	NA
Genotype	α^CD56 (G>A)^α/α^CD98 (TTC>TT‐)^α β^N^/β^N^	α^CD56 (G>A)^α/αα	α^CD98 (TTC>TT‐)^α/αα
HGVS annotation	*HBA2*:c.179G>A/*HBA2*:c.297del	*HBA2*:c.179G>A	*HBA2*:c.297del

*Note:* The normal ranges (adults and paediatrics) for red cell haematology were according to Bain B.J., Bates, I & Laffan, M.A., *Dacie and Lewis Practical Haematology*, 12th Edition. Elsevier 2017.

Abbreviations: CE, capillary electrophoresis; Hb, haemoglobin; HPLC, high performance liquid chromatography; MCH, mean cell haemoglobin; MCHC, mean cell haemoglobin concentration; MCV, mean cell volume; NA; not applicable; RBC, red blood cells; RDW, red cell distribution width.

### 2.2 Latest Review

At her most recent clinical assessment at 3 years and 2 months of age, the proband appeared alert but pale with good perfusion and normal vital signs. Cardiovascular and respiratory examination were unremarkable. Abdominal examination revealed splenomegaly (spleen palpable 8 cm below the left subcostal margin) and justpalpable liver tip. Growth parameters were appropriate for age, with weight (14.4 kg) and height (96 cm) at the 50th percentile on WHO chart. According to her, the child remains active with a good appetite and normal developmental milestones.

### 2.3 Haematological Analysis

A routine blood analysis was performed using an automated cell counter (Sysmex SX‐1000i; Sysmex Co. Ltd., Kobe, Japan). The proband's levels of Hb A, Hb A2, Hb F, and other haemoglobin variants were detected with capillary electrophoresis (Sebia, Evry Cedex, France) and high‐performance liquid chromatography (HPLC).

### 2.4 Molecular Analysis

The DNA was extracted from peripheral blood leukocytes using QIA symphony SP (Qiagen, GmbH, Germany). The concentration and quality of the extracted DNA were measured using NanoDrop 1000 Spectrophotometer (ThermoFisher Scientific Inc., Wilmington, DE, USA). To exclude Hb H disease, two multiplex assays were performed. Multiplex Gap‐PCR assays were used to exclude the most common α‐globin deletions that consist of seven common deletions (−α3.7, −α4.2, and five double gene deletions, ‐‐SEA, ‐‐MED, ‐‐FIL, ‐‐THAI, −(α)20.5) and multiplex ARMS‐PCR targeted six frequent non‐deletional variants using previously described methods [11, 12]. Multiplex ARMS‐PCR detected non‐deletional mutations in the α‐globin gene [initiation codon (*HBA2*:c.2delT), codon 30 (*HBA2*:c.91_93delGAG), codon 35 (*HBA2*:c.106 T>C), Hb Adana (*HBA2*:c.179G>A), Hb Quong Sze (*HBA2*:c.377 T>C) and Hb Constant Spring (*HBA2*:c.427 T>C)] [12]. Based on the first line molecular methods, we found the proband has heterozygous CD 59 (GGC>GAC) [Gly>Asp] *HBA2*:c.179G>A. Furthermore, alpha and beta globin gene sequencing was done to rule out the possibility of a compound heterozygosity for rare alpha and beta‐globin variants.

### 2.5 In Silico Prediction

The structural and functional effects of the *HBA2*:c.297del (p.Phe99Leufs*4) mutation were evaluated using multiple bioinformatics tools. Wild‐type and mutant protein sequences were modelled using SWISS‐MODEL, and visualised with BIOVIA Discovery Studio to assess domain loss and conformational changes [13]. NMDetective was used to predict nonsense‐mediated mRNA decay (NMD) susceptibility based on the location of the premature stop codon [14]. Structural quality was assessed using MolProbity, focusing on clashscore, Ramachandran statistics and backbone geometry [15].

### 2.6 Ethics Approval

This study was conducted in accordance with the 2024 Declaration of Helsinki and was approved by the Medical Research and Ethics Committee (MREC), Ministry of Health, Malaysia. Written informed consent was obtained from the family prior to sample collection. All data were anonymised and confidentiality was ensured through coded identification.

## 3 Results

### 3.1 Haematological Analysis

The MCV, MCH and Hb A2 levels of the proband and her parents are shown in Table 1. In addition, CE and HPLC data revealed in the proband's sample an HbH and Hb Bart's fraction of 0.4% and 7.2% respectively (Figure 1). The Hb A2 was low at 0.7% and Hb F was 34.5% as expected in a two‐month‐old child. HPLC showed similar findings with a low Hb A2 level of 1.3%, a pre‐ calibration peak indicative of the presence of Hb H and Hb Barts with Hb F of 33.2%. No other abnormal variants were detected in either CE or HPLC. These findings are suggestive for the proband to have Hb H disease.

### 3.2 Mutation Analysis of the Thalassaemia Genes

**FIGURE 3:**
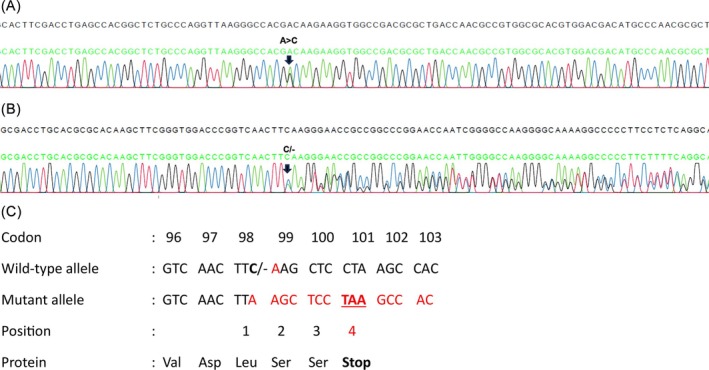
Forward direction nucleotide sequence analysis of the α2 globin gene of the proband (A) Arrow indicates the substitution change G>A at codon 59 (B) Compound heterozygous Hb Adana and Novel Hb A2 variant (*HBA2*: C.297del) as the result of deletion of a single Cytosine at codon 98 causing a frameshift that introduces a premature stop codon (TAA) at position 4. (C) The frameshift mutation occurred at amino acid position 99, where phenylalanine (Phe) is replaced by leucine (Leu), causing a premature stop codon 4 amino acids downstream p.(Phe99Leufs*4).

Screening for common deletional and non‐deletional alpha‐thalassaemia was performed using Gap‐PCR and ARMS‐PCR, which revealed the presence of a heterozygous CD 59 (GGC>GAC) [Gly>Asp]; (*HBA2*:c.179G>A) variant which is the highly unstable Hb Adana in the proband, inherited from her father. Subsequent Sanger sequencing of the *HBA1* and *HBA2* genes identified a compound heterozygosity for Hb Adana and Hb Taiping (*HBA2*:c. 297del) in the proband as shown in Figure 3A–C. Genotyping of the mother who carried the novel *HBA2*:c.297del variant confirmed that it was inherited in trans with Hb Adana in proband. The *HBA2*:c.297del variant was submitted to the IthaGenes database under variant ID 4137. Beta‐globin (*HBB*) gene sequencing in the proband showed a normal genotype.

### 3.3 Classification of the New Variant *HBA2* :C.297del

The *HBA2*:c.297del [p.Phe99fs] variant was annotated using the ACMG/AMP‐specified criteria by the ClinGen Hemoglobinopathy Variant Curation Expert Panel (VCEP), which are currently submitted on the Criteria Specification (CSpec) Editor and pending ClinGen approval [16].

The deletion of a cytosine (C) at position c.297 in codon 98 of exon 3 results in a frameshift and induces a premature stop codon (TAA) at codon 101 [PVS1_S] [17]. This variant is rare, as it is absent from the gnomaDv4.1.0 population database [PM2_P]. In the heterozygous state, it is associated with microcytic hypochromic red cell indices, while, when inherited in trans with the pathogenic Hb Adana variant (c.179G>A, p.Gly60Asp), with phase confirmed, it results in a severe clinical phenotype consistent with the Hb H disease, requiring transfusions from an early age [PM3]. There is evidence to support a likely pathogenic classification, although additional cases or functional data would help to further clarify its clinical impact. The data summaries in Table S1.

### 3.4 Structure Prediction and Visualisation

Multiple bioinformatics tools were employed to evaluate the impact on protein structure, stability, and degradation potential.

**FIGURE 4:**
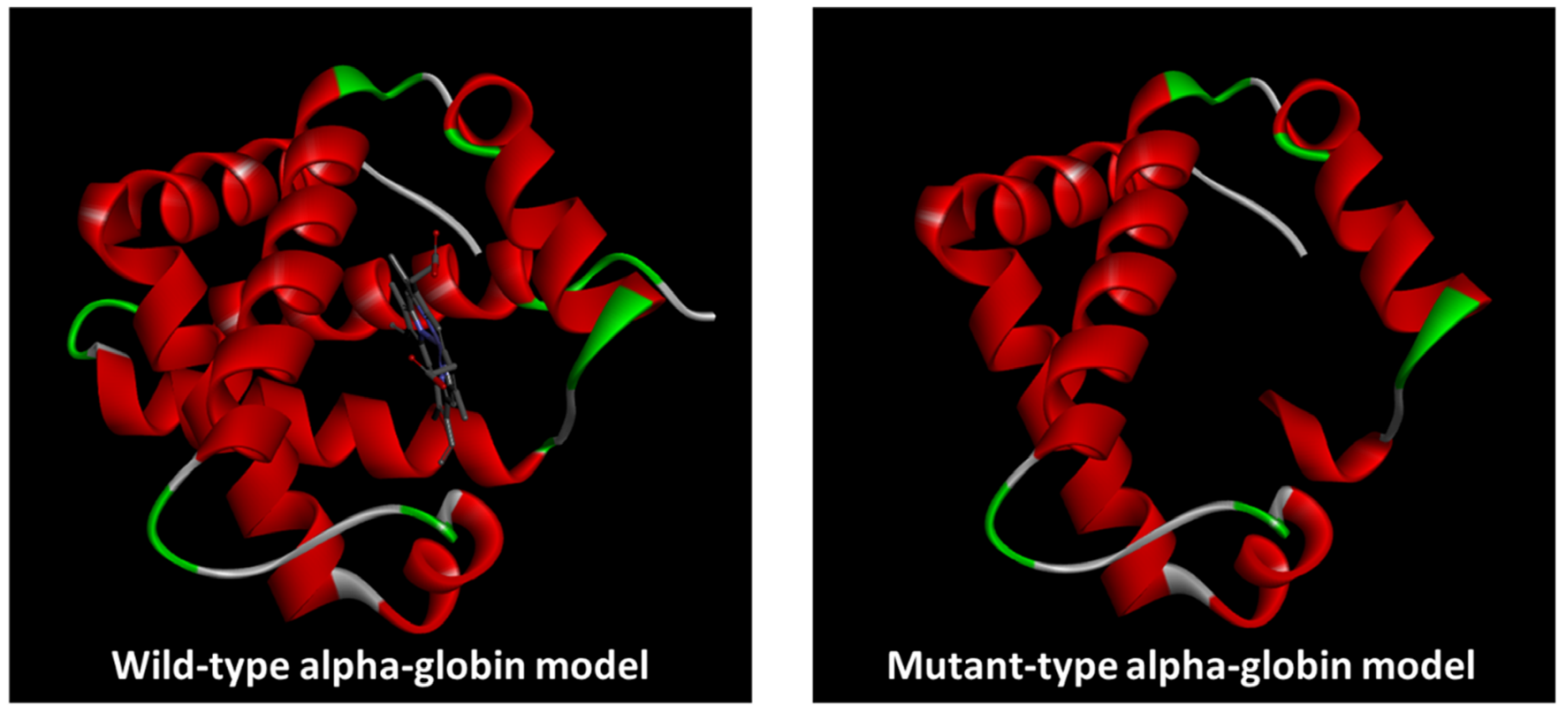
Predicted 3D structures of wild‐type and mutant HBA2 proteins showing loss of C‐terminal helices and heme‐binding pocket in the truncated mutant model.

Using SWISS‐MODEL, three‐dimensional (3D) structures were generated for both the wild‐type (142 amino acids) and mutated (truncated, 101 amino acids) HBA2 proteins. Visualisation via BIOVIA Discovery Studio revealed that the wild‐type alpha‐globin model displayed a complete globin fold with intact α‐helices and a central heme‐binding pocket (Figure 4). In contrast, the mutant model exhibited a truncated structure lacking the C‐terminal helices and the heme‐binding region, indicating significant structural disruption.

### 3.5 Nonsense‐Mediated Decay (NMD) Prediction

The NMDetective tool predicted a low NMD score of 0.12 for the c.297del mutation, suggesting the mutant mRNA is likely to escape NMD degradation and be translated into a truncated protein product.

### 3.6 Structural Quality Assessment (MolProbity)

The wild‐type model exhibited a MolProbity score of 1.51, placing it in the 95th percentile, with no Ramachandran outliers and minimal steric clashes. The mutant model had a numerically superior MolProbity score of 0.72 (100th percentile), but a significantly elevated Ramachandran Z‐score (4.03 ± 0.84) indicated abnormal backbone geometry, likely due to truncation and structural incompleteness.

## 4 Discussion

This case highlights the identification and characterisation of a novel *HBA2* variant associated with α‐thalassaemia, discovered in a Malay family from Taiping, Malaysia. We propose naming this variant Hb Taiping, in recognition of the geographical location of its first discovery. No abnormal haemoglobin peaks were detected on either capillary electrophoresis (CE) or high‐performance liquid chromatography (HPLC), suggesting that both Hb Taiping and Hb Adana are unstable haemoglobin variants—consistent with their rapid degradation in vivo.

The proband's mother, heterozygous for the novel *HBA2* codon 98 deletion (TTC>TT‐), displayed a phenotype consistent with an α^+^ thalassaemia carrier, with only mild microcytic hypochromic anaemia. In contrast, the proband exhibited a significantly more severe clinical phenotype, explained by compound heterozygosity for the novel variant and Hb Adana (*HBA2*:c.179G>A), confirmed to be present in trans. The absence of significant β‐globin variants on full gene sequencing supports the conclusion that the severe phenotype is solely due to α‐globin pathology.

Hb Adana is a well‐known highly unstable α‐globin variant caused by a missense variant at codon 59, most frequently occurring in the *HBA2* gene in Southeast Asian populations, including Malaysia and Indonesia [18, 19]. In contrast, the α1‐globin gene variant is more commonly reported among Turkish and Middle Eastern populations [20]. Numerous studies have described the interaction of Hb Adana with both deletional and non‐deletional α‐thalassaemia mutations. When inherited in trans with non‐deletional variants such as Hb Constant Spring, Hb Quong Sze, or Hb Pakse, the resulting compound heterozygous states frequently lead to Hb H disease, with some progressing to transfusion‐dependent thalassaemia (TDT) [4, 21]. Most cases present during infancy or early childhood, as seen in our proband.

To our knowledge, this is the first report of the novel *HBA2* codon 98 deletion causing a premature stop codon, resulting in a truncated α‐globin chain. The predicted impact of this variant aligns with ACMG/AMP criteria for pathogenicity, including the occurrence in trans with known pathogenic variants and manifest severe intermediate phenotype (PM3), consistent segregation with disease phenotype within the family and absence from population databases such as gnomAD and dbSNP (PM2_P) and in silico tools predicting loss of function (PVS1).

Based on in silico analysis, the frameshift mutation c.297del in HBA2 (p.Phe99Leufs*4) results in a truncated alpha‐globin that lacks key structural motifs critical for heme binding and tetramerisation. The NMDetective prediction (score = 0.12) suggests the mutant mRNA likely escapes nonsense‐mediated decay, enabling translation of the aberrant protein. Structural evaluation using MolProbity revealed an elevated Ramachandran Z‐score, indicating backbone strain despite acceptable all‐atom geometries. Overall, the data indicate that c.297del induces a pathogenic mechanism through haploinsufficiency, where a misfolded and nonfunctional protein accumulates without effective heme incorporation, contributing to alpha thalassaemia. This study further highlights the value of integrated in silico approaches, which contribute structural validation, thermodynamic profiling, and transcript degradation prediction to investigate variant pathogenicity.

Globally, more than 1800 haemoglobin variants have been reported in databases such as HbVar and IthaGenes. However, only a small number involve nonsense mutations or frameshifts that introduce premature stop codons in either *HBA1* or *HBA2*, underscoring the rarity and clinical importance of this finding. The addition of Hb Taiping expands the known mutational spectrum of α‐thalassaemia and highlights the continuing need for precise molecular characterisation in atypical cases. This case underscores the importance of integrating detailed haematological, molecular, and bioinformatic analyses for accurate diagnosis and variant classification. Early identification and proper classification of such pathogenic variants are essential for effective genetic counselling, family screening, and prenatal diagnostic strategies.

## 5 Conclusion

This report underscores the importance of adapting ACMG/AMP guidelines for accurate classification of globin gene variants. The identification of a novel *HBA2* variant, Hb Taiping, in trans with Hb Adana and associated with a severe phenotype illustrates the value of integrating molecular, clinical, and computational data in variant interpretation. Haemoglobinopathies pose unique diagnostic challenges due to genetic complexity and phenotypic variability. Applying tailored ACMG/AMP criteria enables standardised, evidence‐based classification, which is essential for accurate diagnosis, genetic counselling, and clinical management. Our findings contribute to the growing body of curated haemoglobin variants and highlight the need for continued refinement of variant interpretation frameworks specific to globin genes, particularly in high‐prevalence regions like Southeast Asia.

Importantly, this case also highlights the critical role of carrier screening and genetic counselling in preventing severe haemoglobinopathies. Standard screening panels may fail to detect rare or novel non‐deletional mutations, as initially occurred in the mother. This reinforces the value of sequencing in cases with discordant clinical or haematological findings and the necessity of expanding screening strategies to include both common and rare variants in endemic areas. Early and accurate identification of at‐risk couples is essential for informed reproductive choices, timely diagnosis, and appropriate clinical follow‐up.

## Author Contributions

N.M.Y., T.K., C.L.H. performed study concept and design, development of methodology and writing, review and revision of paper. N.M.Y., S.S., F.S.A.H., S.H., E.S.Z., N.N.M., N.A.A., C.L.H. were involved in analysis and interpretation of data. C.S., P.K., S.J.G.A., T.K., N.M.Y., T.P. performed curation of the variant. M.N.D. was involved in construction of protein model. C.M.L. and N.N.M. provided the details clinical history. All authors read and approved the final paper.

## Funding

This research is supported by COST (European Cooperation in Science and Technology).

## Conflicts of Interest

The authors declare no conflicts of interest.

## Supporting information


**Table S1:** ClinGen haemoglobinopathy VCEP‐specified ACMG/AMP criteria.

## Data Availability

The data that support the findings of this study are available on request from the corresponding author. The data are not publicly available due to privacy or ethical restrictions.
