# Supplementary material for: ACMG/AMP‐Based Variant Classification of a Novel HBA2 Variant ( HBA2 : C.297del, Hb Taiping) in Compound Heterozygosity With Hb Adana ( HBA2 :C.179G>A) Causing Non‐Deletional Hb H Disease
Source: Int J Lab Hematol. 2026 Jan 6;48(2):458–65. doi: 10.1111/ijlh.70037 (PMC12956499; doi:10.1111/ijlh.70037)
Supplement: Supplementary file 1 — Table S1: ClinGen haemoglobinopathy VCEP‐specified ACMG/AMP criteria. [file IJLH-48-458-s001.docx]

Table S1: ClinGen haemoglobinopathy VCEP-specified ACMG/AMP criteria

| Criteria | Justification |
| --- | --- |
| PVS1_S | LoF variant in gene where LoF causes disease |
| PM2_P | Absent/rare in population databases (e.g., AF < 0.0001) |
| PM3 | Detected in trans with known pathogenic variant; consistent with recessive disease |
